# Associations Between Endocrine-Disrupting Chemical Exposure and Fertility Outcomes: A Decade of Human Epidemiological Evidence

**DOI:** 10.3390/life15070993

**Published:** 2025-06-21

**Authors:** Zoe Tzouma, Panagiota Dourou, Athina Diamanti, Vikentia Harizopoulou, Petros Papalexis, Grigorios Karampas, Alina Liepinaitienė, Audrius Dėdelė, Antigoni Sarantaki

**Affiliations:** 1 Midwifery Department, Faculty of Health & Care Sciences, University of West Attica, Egaleo, 12243 Athens, Greeceadiamanti@uniwa.gr (A.D.); vharizopoulou@uniwa.gr (V.H.); 2 Unit of Endocrinology, 1st Department of Internal Medicine, Laiko General Hospital, National and Kapodistrian University of Athens, 11527 Athens, Greece; petranpapalex@gmail.com; 3 2nd Department of Obstetrics and Gynecology, Aretaieio University Hospital, Medical School, National and Kapodistrian University of Athens, 11528 Athens, Greece; karampasgrig@gmail.com; 4 Department of Obstetrics and Gynecology, Scänes University Hospital, 21428 Malmö-Lund, Sweden; 5 Department of Environmental Sciences, Faculty of Natural Sciences, Vytautas Magnus University, LT-44404 Kaunas, Lithuania; alinute91@gmail.com (A.L.); audrius.dedele@vdu.lt (A.D.); 6 Kauno Kolegija Higher Education Institution, Faculty of Medicine, Pramonės av. 20, LT-50468 Kaunas, Lithuania

**Keywords:** endocrine-disrupting chemicals, reproductive health, infertility, hormonal imbalance, bisphenol A

## Abstract

Endocrine-disrupting chemicals (EDCs) are exogenous compounds that interfere with the endocrine system by mimicking or blocking the action of endogenous hormones such as estrogens, androgens, and thyroid hormones. This systematic review aims to evaluate the current epidemiological evidence linking EDC exposure with adverse reproductive outcomes in males and females of reproductive age. A total of 14 observational studies published between 2014 and 2024 were included following structured searches in PubMed, Scopus, and Google Scholar. The most commonly studied EDCs included bisphenol A (BPA), its analogs (such as bisphenol S, BPS), phthalates, parabens, per- and polyfluoroalkyl substances (PFAS), and persistent organic pollutants (POPs). The review found consistent associations between EDC exposure and multiple reproductive endpoints, such as impaired semen quality, decreased ovarian reserve, infertility, polycystic ovary syndrome (PCOS), altered hormone levels—specifically estradiol (E2), luteinizing hormone (LH), and follicle-stimulating hormone (FSH)—and adverse outcomes in assisted reproductive technologies (ART), including in vitro fertilization (IVF). Despite methodological heterogeneity, the findings support the biological plausibility of EDCs in disrupting reproductive function. The review highlights the urgent need for regulatory measures, increased public awareness, and longitudinal studies to assess the cumulative effects of chronic EDC exposure on human fertility.

## 1. Introduction

Endocrine-disrupting chemicals (EDCs) are exogenous substances that can interfere with the normal functioning of the endocrine system by mimicking, blocking, or altering the synthesis, transport, metabolism, or elimination of endogenous hormones such as estrogens, androgens, and thyroid hormones. These hormonal perturbations can have profound effects on numerous physiological systems, particularly those governing reproductive health and development [1,2]. The endocrine system is especially sensitive during critical windows of vulnerability, such as fetal development, puberty, and early adulthood, rendering individuals exposed during these periods particularly susceptible to long-term consequences [1].

EDCs comprise a structurally diverse group of compounds, including both naturally occurring and synthetic agents. Natural EDCs include phytoestrogens, whereas synthetic variants encompass industrial and consumer product-related substances such as polychlorinated biphenyls (PCBs), phthalates, bisphenol A (BPA), dioxins, organochlorine pesticides like dichlorodiphenyltrichloroethane (DDT), and a broad class of fluorinated compounds known as per- and polyfluoroalkyl substances (PFAS). These chemicals are prevalent in everyday materials and consumer products, including plastics, food packaging, household dust, detergents, cosmetics, personal care products, and children’s toys. Consequently, human exposure to EDCs is both widespread and continuous, occurring through various routes, including ingestion, inhalation, and dermal absorption [3,4].

In recognition of the potential health threat posed by EDCs, the World Health Organization (WHO) and the United Nations Environment Programme (UNEP) jointly released a pivotal report in 2012 titled State of the Science of Endocrine Disrupting Chemicals. This report provided a comprehensive synthesis of the scientific evidence at the time. It emphasizes that EDCs can interfere with hormonal signaling, impair tissue and organ development, and increase the risk of various non-communicable diseases. Of particular concern were the documented effects on fertility and reproductive function, including altered puberty onset, reduced fecundity, impaired gametogenesis, and increased risk of reproductive system abnormalities [5].

Since the release of the WHO report, there has been growing research interest in the reproductive health implications of EDCs. However, despite a rising number of studies and advancements in analytical chemistry and epidemiological methods, substantial knowledge gaps persist. Key unresolved issues include the effects of chronic low-dose exposure over time, the synergistic or antagonistic interactions between multiple EDCs (often referred to as the “cocktail effect”), and the lack of consensus on threshold levels that can be considered safe or tolerable for human health [6]. Moreover, many EDCs are lipophilic and persistent in the environment, raising concerns about their bioaccumulation and transgenerational effects [6].

Recent epidemiological studies have increasingly shown statistically significant associations between EDC exposure and various adverse reproductive outcomes in both sexes. These include reductions in sperm count, motility, and morphology; increased infertility rates; hormonal dysregulation; and a heightened prevalence of reproductive disorders such as polycystic ovary syndrome (PCOS), endometriosis, and diminished ovarian reserve [7,8,9,10]. Furthermore, EDCs have been implicated in compromising outcomes in assisted reproductive technologies, including in vitro fertilization (IVF), and may adversely influence pregnancy maintenance and fetal development.

Nevertheless, the current body of literature is marked by considerable heterogeneity in study designs, populations, analytical techniques for chemical exposure measurement, and outcome definitions. These inconsistencies limit the comparability of findings and hinder the ability to draw robust, generalizable conclusions. Additionally, the majority of human studies remain observational in nature, and often face challenges related to confounding, reverse causation, and exposure misclassification.

Given the growing global concern surrounding the decline in fertility rates and reproductive capacity, alongside the mounting burden of environmental exposures, there is a critical need for a structured and comprehensive evaluation of the existing evidence. The present systematic review aims to address this need by synthesizing data from observational studies that investigate the relationship between exposure to hormone-mimicking EDCs and adverse reproductive health outcomes in males and females of reproductive age. Through an integrated analysis of current findings, this review seeks to clarify the reproductive impairments most consistently associated with EDC exposure, explore the underlying biological mechanisms, identify key limitations in the current research landscape, and provide targeted recommendations for future studies and public health policy development.

## 2. Materials and Methods

The study was conducted according to the Preferred Reporting Items for Systematic Reviews and Meta-Analyses (PRISMA) guidelines [11]. This systematic review has been registered in the International Prospective Register of Systematic Reviews (PROSPERO) with ID number CRD420251021592.

### 2.1. Search Strategy

Searches for studies were conducted in PubMed, Scopus, and Google Scholar. The search included studies published in English between December 2014 and December 2024, focusing on free full-text, peer-reviewed articles related to endocrine disruptors and fertility.

The Boolean search strings used were tailored to each database:

PubMed: (“Endocrine Disrupting Chemicals”[Mesh] OR “EDCs” OR “Persistent Organic Pollutants” OR “Hormone Mimic” OR “Bisphenol A”[Mesh] OR “Phthalates”[Mesh] OR “Dioxins”[Mesh] OR “Pesticides”[Mesh]) AND (“Fertility”[Mesh] OR “Infertility”[Mesh] OR “Reproductive Health”[Mesh] OR “Sperm Quality” OR “Ovarian Function”) AND (“2014/12/01”[Date-Publication]: “2024/12/01”[Date-Publication]) AND (“Humans”[Mesh]) AND English[lang]

Scopus: TITLE-ABS-KEY (“endocrine disrupting chemicals” OR “EDCs” OR “persistent organic pollutants” OR “hormone mimic” OR “bisphenol A” OR “phthalates” OR “dioxins” OR “pesticides”) AND TITLE-ABS-KEY(“fertility” OR “infertility” OR “reproductive health” OR “sperm quality” OR “ovarian function”) AND LIMIT-TO(LANGUAGE, “English”) AND PUBYEAR > 2013 AND PUBYEAR < 2025

Google Scholar: allintitle: (“endocrine disrupting chemicals” OR “EDCs” OR “persistent organic pollutants” OR “hormone mimic” OR “bisphenol A” OR “phthalates” OR “dioxins” OR “pesticides”) AND (“fertility” OR “infertility” OR “reproductive health” OR “sperm quality” OR “ovarian function”) after: 2013 before: 2025

### 2.2. Inclusion and Exclusion Criteria

This review included peer-reviewed clinical trials, cohort studies, and case–control studies that investigated the effects of hormone-mimicking endocrine-disrupting chemicals (EDCs) on fertility outcomes in males or females of reproductive age. Eligible studies were required to report specific reproductive health metrics (e.g., sperm quality, ovarian reserve, infertility rates) and provide direct measures or estimates of exposure to hormone-mimicking EDCs.

Studies were excluded if they (i) involved animal or in vitro models; (ii) were not peer-reviewed, with the exception of relevant gray literature such as governmental or institutional reports; or (iii) did not present explicit reproductive outcomes or failed to assess EDC exposure directly.

### 2.3. PRISMA Process

A total of 9578 records were initially identified through database searches. Before screening, 18 duplicate records were removed, and 9428 records were excluded by automation tools due to ineligibility, leaving 132 records for initial screening. During the screening phase, 11 records were excluded based on their titles and abstracts due to irrelevance to the research question. The remaining 121 full-text articles were retrieved and assessed for eligibility. Upon full-text review, 107 records were excluded for the following reasons: 20 were not primary research studies, 43 did not investigate EDC exposure or reproductive outcomes, 15 involved animal models, and 29 were deemed methodologically weak or lacked sufficient data quality. Ultimately, 14 studies met all inclusion criteria and were included in the final systematic review.

**Figure 1:**
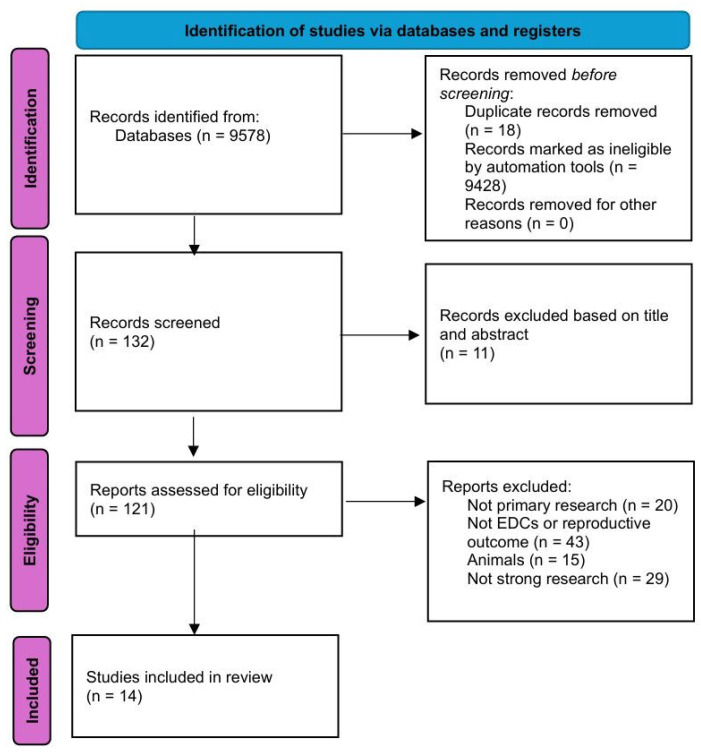
Study selection process.

The study selection process is illustrated in Figure 1.

### 2.4. Risk of Bias Assessment

The risk of bias and methodological quality of the included studies were evaluated using the framework proposed by Caldwell et al. [12], which is specifically designed for the appraisal of observational research. This tool examines several critical domains, including the clarity and relevance of the research question, appropriateness of the study design, comparability of study populations, precision and validity of exposure and outcome measurements, consideration and control of confounding variables, and the overall transparency of conclusions and data reporting.

Two independent reviewers conducted the assessments for each study. Discrepancies were resolved through discussion, and consensus was reached prior to assigning final quality ratings. A detailed summary of the quality assessments is provided in Supplementary Table S1.

### 2.5. Data Extraction

A standardized data extraction form was developed using Microsoft Excel to ensure consistency and comprehensiveness across all included studies. The following information was systematically extracted from each study: author(s), year of publication, country of study, study design and sample size, methods used for exposure and outcome measurement, presence and characteristics of comparison groups, specific reproductive health outcomes assessed, principal findings, fertility-related results, and any reported follow-up periods or study limitations.

## 3. Results

Fourteen studies were included in this systematic review [9,10,13,14,15,16,17,18,19,20,21,22,23,24]: six case–control, five cohort, and three cross-sectional studies. These studies were conducted in China (seven), the USA (four), and one each in Poland, Jordan, and Iran.

A summary of the individual study characteristics and findings is provided in Table 1. The included studies investigated a range of endocrine-disrupting chemicals, including phthalates, bisphenol A and its analogs, parabens, triclosan, persistent organic pollutants (POPs), and pesticides. They explored associations with various reproductive outcomes such as sperm quality, PCOS, ovarian reserve, IVF success, pregnancy outcomes, and hormone levels. Overall, the studies consistently reported associations between EDC exposure and adverse reproductive health outcomes in both sexes. Most case–control and cohort studies found higher levels of EDCs in cases than in controls, and several identified dose–response relationships. Outcomes included reduced sperm motility and concentration, increased risk of PCOS, infertility, spontaneous abortion, decreased ovarian reserve, poorer embryo quality in IVF, altered hormone levels (estradiol, E2; luteinizing hormone, LH; follicle-stimulating hormone, FSH), and earlier menopause.

These results support the hypothesis that exposure to hormone-mimicking endocrine disruptors is linked with measurable and clinically significant effects on human fertility and reproductive health.

### 3.1. Male Reproductive Outcomes

Five studies assessed the impact of EDC exposure on male reproductive parameters. Ramsay et al. [13], utilizing a large retrospective cohort of subfertile men in the United States, reported a significant inverse relationship between airborne industrial EDC exposure and semen analysis parameters, including motility, concentration, and ejaculate volume. Similarly, Gao et al. [21] observed that high urinary levels of EDCs were negatively associated with sperm motility and, to a lesser extent, sperm concentration among men undergoing IVF therapy. Palak et al. [24] demonstrated that BPA exposure was associated with adverse steroid hormone profiles and decreased sperm quality, including morphology and concentration. These findings collectively support the hypothesis that EDC exposure may impair spermatogenesis and hormonal regulation. Additional indirect evidence from Rahimi et al. [22] suggested that occupational pesticide exposure among women may also be linked to male-mediated reproductive outcomes such as spontaneous abortion and infertility.

### 3.2. Female Reproductive Outcomes

Eight studies focused on female reproductive disorders and fertility outcomes. Wei et al. [14] found significantly higher serum levels of EDCs, including parabens, bisphenols, TCS, and phthalate metabolites, among women diagnosed with infertility compared to controls. Abdo et al. [18] reported elevated levels of di(2-ethylhexyl) phthalate (DEHP) metabolites in infertile women, suggesting a positive association between phthalate exposure and female sterility. Pan et al. [19] demonstrated that increased blood levels of POPs, particularly para, para′-Dichlorodiphenyltrichloroethane (p, p′-DDT), and PCBs, were associated with an elevated risk of primary ovarian insufficiency (POI). Zhan et al. [9] reported significantly higher concentrations of BPA and its analogs (bisphenol S, BPS; bisphenol P; BPP, bisphenol Z, BPZ; bisphenol AF, BPAF) in women diagnosed with PCOS, suggesting a role of bisphenol exposure in endocrine-related ovarian dysfunction. Similarly, Zhang et al. [10] found that serum BPS and BPA levels were significantly associated with decreased ovarian reserve (DOR), as reflected by altered levels of anti-Müllerian hormone (AMH), E2, and FSH.

### 3.3. IVF and Early Reproductive Outcomes

Three studies specifically investigated IVF and early reproductive outcomes. Zeng et al. [15] found that elevated levels of PFAAs in follicular fluid were associated with poorer embryo quality among women undergoing IVF. Li et al. [23] observed that higher concentrations of EDCs in follicular fluid correlated with adverse early reproductive indicators, including reduced numbers of mature and fertilized oocytes and lower-quality embryos. Zhang et al. [17] identified a significant association between prenatal exposure to BPA, BPS, benzophenone-3, and TCS with the risk of preterm birth. Together, these findings suggest that EDC exposure may interfere with key processes of assisted reproduction and early pregnancy maintenance.

### 3.4. Hormonal and Biomarker Disruptions

Two studies focused on hormonal and systemic biomarkers of reproductive health. Nobles et al. [16] demonstrated that phthalate exposure was associated with hormonal imbalances (decreased E2, increased LH and FSH at ovulation), inflammation, and oxidative stress among women attempting to conceive. Pollack et al. [20] found that exposure to BPA, chlorophenols, benzophenones, and parabens was associated with altered ovarian hormone levels, including elevated E2 and progesterone, and reduced FSH and LH levels. These biomarker studies provide mechanistic insights into how EDCs may disrupt the hypothalamic–pituitary–gonadal axis.

## 4. Discussion

This systematic review consolidated evidence from 14 observational studies that explored the link between exposure to hormone-mimicking EDCs and reproductive health outcomes in males and females of reproductive age.

The findings consistently demonstrate that EDCs—including BPA and its analogs, phthalates, parabens, per- and PFAS, PCBs, and persistent organic pollutants such as DDT—are significantly associated with a broad range of adverse reproductive outcomes. In males, exposure to these chemicals was linked to reduced sperm quality, including motility, morphology, and concentration [13,21,24]. In females, associations were observed with infertility, DOS, hormonal imbalance, and endocrine-related disorders such as PCOS and POI [9,10,14,18,19]. Several studies also reported associations between EDC exposure and impaired outcomes in assisted reproductive technologies (ART), such as lower embryo quality and increased risk of preterm birth [15,17,23]. Additionally, systemic biomarkers of reproductive function, such as E2, FSH, and LH, were found to be disrupted after exposure to various EDCs [16,20], further supporting the mechanistic plausibility that endocrine disruption leads to reproductive dysfunction.

The results align with previous reports. The review by Di Nisio and Foresta [25] comprehensively examines how EDCs such as phthalates, bisphenol A (BPA), organophosphate pesticides (OPs), perfluoroalkyl compounds (PFCs), and cadmium (Cd) adversely affect male fertility through multiple mechanisms. These chemicals mimic or antagonize natural hormones, disrupt steroidogenesis, impair hypothalamic–pituitary–gonadal axis regulation, and interfere with androgen and estrogen receptors, leading to reduced testosterone production, altered sperm parameters (count, motility, morphology), and increased sperm DNA damage. Phthalates and BPA were strongly linked to anti-androgenic effects and poor semen quality, while cadmium exhibited consistent testicular toxicity and hormonal disruption in experimental models. Although epidemiological studies show varying degrees of association due to methodological differences, geographic factors, and population heterogeneity, the overall evidence supports a positive link between EDC exposure and male reproductive dysfunction [25].

The review by Presunto et al. [26] specifically focused on BPA and its effects on human male infertility, highlighting both epidemiological evidence and mechanistic pathways. BPA, a widely used endocrine-disrupting chemical, mimics or antagonizes endogenous hormones, primarily by binding to estrogen receptors (ERα and ERβ), androgen receptors, G-protein-coupled estrogen receptors (GPER), and other nuclear receptors like PPAR-γ and ERR-γ. It disrupts the hypothalamic–pituitary–gonadal axis, alters gene expression involved in testicular steroidogenesis, and increases oxidative stress by reducing antioxidant defense, leading to DNA damage in sperm. These mechanisms collectively impair spermatogenesis and reduce semen quality. Additionally, BPA can inhibit DNA methylation and induce epigenetic changes, including alterations in microRNA expression, which may further disrupt testicular function and contribute to transgenerational reproductive effects. While animal models consistently show a detrimental effect of BPA on male fertility, human epidemiological studies reveal mixed results, though many link BPA exposure to altered hormone levels (e.g., FSH, LH, testosterone) and deteriorated sperm parameters. Despite some inconsistent findings, the cumulative evidence suggests a significant risk of BPA-induced male reproductive dysfunction through hormonal, oxidative, and epigenetic pathways.

The review by Asori et al. [27] explored the impact of endocrine-disrupting chemicals (EDCs) on reproductive health, with a particular focus on those found in personal care and beauty products, highlighting their role in male and female infertility. The authors systematically reviewed evidence showing that EDCs such as phthalates, bisphenol A (BPA), parabens, dioxins, and methoxychlor—commonly present in cosmetics, soaps, and plastic products—can impair reproductive function through hormonal interference, oxidative stress, and receptor modulation. In males, these chemicals are linked to sperm DNA damage, reduced sperm count and motility, testicular anomalies, and alterations in steroidogenesis, while in females, they are associated with ovarian dysfunction, premature ovarian failure, endometrial abnormalities, fibroid development, and structural damage to the uterus and vagina. The review emphasized the need for spatial and demographic considerations in risk assessments, noting variations in exposure and effects across different populations, and calls for increased public awareness, research funding, and regulatory oversight to mitigate these risks.

The review by Amir et al. [28] provided an in-depth analysis of how EDCs such as BPA, PCBs, DDT, and phthalates interfere with reproductive health by acting primarily through estrogen and androgen pathways. These EDCs mimic or block natural hormones by binding to ERα and ERβ and androgen receptors (AR), functioning as agonists or antagonists and thereby disrupting gene transcription and hormonal signaling. Mechanistically, they affect reproductive function through various pathways, including receptor-mediated transcription, activation of alternative receptors such as GPCR and ERRγ, and disruption of intracellular signaling cascades.

EDCs also cause significant genomic and epigenomic alterations such as chromosomal aberrations, DNA damage via oxidative stress, and epigenetic modifications including abnormal DNA methylation, histone changes, and altered microRNA expression. These disruptions impair spermatogenesis, ovarian development, and hormonal balance, often resulting in infertility in both sexes and transgenerational reproductive defects.

Beyond these epidemiological associations, several biological mechanisms have been suggested to explain how endocrine-disrupting chemicals (EDCs) might negatively impact human fertility. A key pathway involves the disruption of the hypothalamic–pituitary–gonadal (HPG) axis, where compounds like bisphenol A (BPA) and certain phthalates act as agonists or antagonists to estrogen and androgen receptors. This interaction leads to changes in the secretion of gonadotropins, such as luteinizing hormone (LH) and follicle-stimulating hormone (FSH), thereby disrupting processes like folliculogenesis and spermatogenesis. Additionally, epigenetic changes, including DNA methylation and histone modifications—particularly from exposure to phthalates and polychlorinated biphenyls (PCBs)—have been linked to impaired gametogenesis and potential intergenerational effects [29,30].

Oxidative stress and inflammation are central to the reproductive toxicity associated with endocrine-disrupting chemicals (EDCs). Studies have demonstrated that bisphenol A (BPA) and per- and polyfluoroalkyl substances (PFAS) elevate levels of reactive oxygen species (ROS), which in turn cause damage to sperm membranes, decrease motility, and impair oocyte quality. Moreover, exposure to EDCs has been linked to mitochondrial dysfunction in germ cells, indicating a reduction in ATP availability, a critical factor for successful fertilization and early embryonic development. These mechanistic insights provide a biological explanation for the observed clinical outcomes and emphasize the complex nature of endocrine disruption in reproductive health [31,32,33,34].

The present review contributes further by incorporating more recent studies and including emerging chemical classes, such as BPA analogs (e.g., BPS, BPF, BPAF) and PFAS, which have received increased attention in the past few years but were not widely assessed in earlier reviews. The implications of these findings are significant for public health and environmental policy. The ubiquitous presence of EDCs in food packaging, personal care products, plastics, and industrial materials results in near-constant human exposure through ingestion, inhalation, and dermal absorption. The observed associations between EDC exposure and critical fertility-related endpoints highlight an urgent need for policy interventions aimed at exposure reduction. Regulatory actions, such as limiting the use of specific high-risk chemicals in consumer products, enhancing labeling requirements, and promoting safer alternatives, may reduce the cumulative burden of EDCs on reproductive health. Furthermore, public awareness campaigns could empower individuals to make informed choices about product use and minimize avoidable exposures [35,36].

It is important to interpret the findings of this review in the context of potential confounding variables that may have influenced the observed associations between EDC exposure and reproductive outcomes. Most included studies employed multivariable regression models to adjust for known covariates; however, the extent and consistency of adjustment varied. Lifestyle-related confounders such as diet (particularly intake of processed and packaged foods), physical activity, smoking, and alcohol consumption can significantly influence both EDC exposure and fertility outcomes. Occupational exposures are another major source of variability, as individuals working in industries such as plastics, cosmetics, agriculture, or manufacturing may be exposed to substantially higher levels of EDCs compared to the general population. Additionally, socioeconomic status (SES) may impact both exposure risk and access to reproductive care or fertility interventions [37,38,39].

Moreover, underlying health conditions such as obesity, thyroid dysfunction, or metabolic syndrome can independently influence reproductive hormones and fertility, while also affecting susceptibility to endocrine disruption [40]. Many of the studies included did not thoroughly account for these factors, and some lacked longitudinal designs that could better establish temporality. Consequently, the possibility of residual or unmeasured confounding persists, and findings should be interpreted with appropriate caution. Future research should aim to incorporate more robust control of these variables, potentially through stratified analyses, matched study designs, or biomarker-standardized exposure assessments [41].

Despite the consistency of observed associations, this review also reveals important limitations in the existing body of evidence. One of the main challenges lies in the heterogeneity of the included studies. Differences in study designs, population characteristics, chemical exposure assessments (e.g., type of biological sample, timing of collection, detection methods), and outcome measurements hinder direct comparability and preclude formal meta-analysis. Most studies relied on single-point exposure measurements, which may not capture chronic or cumulative exposure levels accurately. Furthermore, the observational nature of nearly all included studies limits causal inference, as unmeasured confounding or reverse causality cannot be ruled out. Additionally, relatively few studies accounted for mixtures of EDCs, despite evidence suggesting that combined exposures may have synergistic or antagonistic effects.

Nevertheless, this review has several methodological strengths. It includes a comprehensive and up-to-date synthesis of studies from multiple countries, offering broad geographical representation and enhancing the external validity of the findings. The use of a structured and transparent selection process, rigorous quality appraisal, and independent data extraction by multiple reviewers strengthens the reliability of the conclusions. Moreover, the review highlights a range of reproductive endpoints, including emerging biomarkers and outcomes relevant to modern reproductive medicine, such as embryo quality and hormonal profiles.

To address current knowledge gaps, future research should prioritize longitudinal cohort studies that can better characterize the temporal relationship between EDC exposure and reproductive outcomes. Such studies should incorporate repeated exposure measurements to reflect internal chemical burden over time and employ standardized, validated protocols for both exposure and outcome assessments. Investigating the mechanisms of EDC action through integrated clinical and experimental designs—including endocrine, genomic, and epigenetic pathways—will be crucial in elucidating causal pathways. Furthermore, research should increasingly focus on the health effects of chemical mixtures to better reflect real-life exposures. Studies evaluating interventions to reduce EDC exposure in vulnerable populations, particularly during pregnancy or preconception, would also provide valuable insight into feasible risk-reduction strategies.

**Figure 2:**
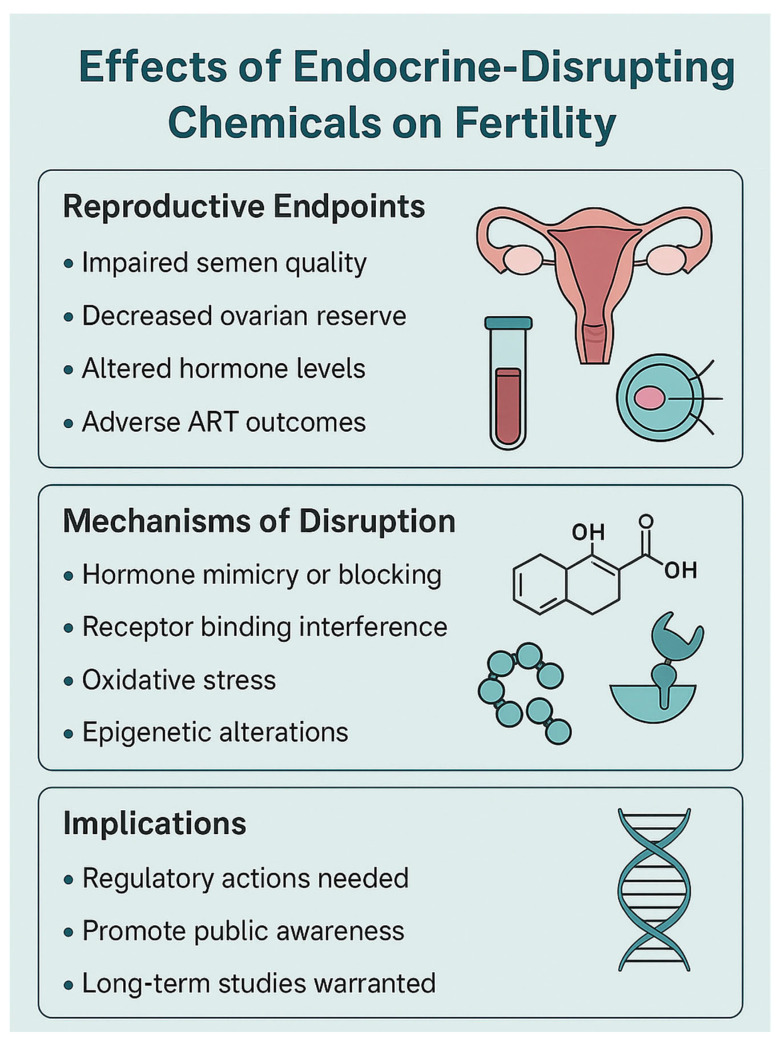
Effects of EDCs on fertility, the underlying mechanisms, and clinical implications. ART: Assisted Reproductive Technologies.

Figure 2 summarizes the effects of EDCs on fertility, the underlying mechanisms, and the clinical implications.

## 5. Conclusions

This systematic review provides compelling evidence that exposure to hormone-mimicking EDCs is consistently associated with a spectrum of adverse reproductive health outcomes in both males and females of reproductive age. The included studies reveal associations between EDCs—particularly BPA, phthalates, parabens, PFAS, and PCBs—and reduced sperm quality, hormonal dysregulation, infertility, PCOS, DOR, POI, and compromised IVF outcomes. These findings reinforce the mechanistic plausibility of endocrine disruption and underscore the widespread risk posed by chronic, low-dose exposure to environmental EDCs. Despite heterogeneity in exposure measurements and study designs, the overall direction of associations was consistent. Continued research is essential to elucidate dose–response relationships, assess long-term and transgenerational effects, and explore intervention strategies. From a public health standpoint, these results advocate for stricter regulatory policies, enhanced product labeling, and preventive strategies to reduce EDC exposure in vulnerable populations. Addressing these environmental threats is vital for safeguarding reproductive health in current and future generations.

## Figures and Tables

**Table 1:** Characteristics of the included studies.

Authors	Year	Country	Type of Study	Participants/Focus Group	Measurement and Data Collection	Comparison Group	Measured Outcome	Key Findings	Specific Fertility Findings	Follow-Up/Study Limitations
Ramsay et al. [13]	2023	U.S.A.	Retrospective cohort study	21,563 men dealing with fertility problems (23,922 samples)	SHARE cohort (all semen analysis results 1996–2017 in Utah), Utah Population Database (UPDB)	Reproductive-age men without fertility problems	Relationship between environmental exposure to airborne EDCs from industrial sources and semen analysis (SA) parameters	EDCs are negatively associated with sperm’s motility, concentration, and ejaculate volume	Increased exposure is associated with higher risk of azoospermia and/or decreased sperm motility	12 years follow-up, limited generalizability due to the sample of infertile men, no information about the occupation, no adjustment for any comorbid medical conditions, lack of diversity
Zhan et al. [9]	2023	China	Case–control study (multi-center, hospital-based)	733 women (321 women with PCOS)	Questionnaires, blood, urine, semen samples	412 women without reproductive or endocrine disorders but with fertility problems due to male infertility	The association of bisphenol A and its analogs with PCOS	Concentrations of bisphenol analogs were significantly higher in the cases group	Increased levels of BPA, BPS, BPP, BPZ and BPAF are associated with high risk of PCOS	2 years follow-up, exposure to BPA was measured after the diagnosis of PCOS, bisphenol analogs have rapid elimination from the body and short half-lives, no information on dietary habits (healthy diet can reduce the odds of PCOS)
Wei et al. [14]	2024	China	Case–control study	302 women (181 with fertility problems)	Blood samples, clinical diagnosis	121 women without fertility problems	Relationship between EDCs (parabens, paraben metabolites, TCS, TCC, bisphenols, benzophenones, phthalate metabolites) and female infertility	Higher levels of EDCs in serum samples of infertile women	EDCs are significantly associated with female infertility	Lack of rigorous inclusion criteria, single sample measurement, need for animal experiments to reinforce the findings
Zeng et al. [15]	2023	China	Prospective cohort study	729 women in IVF treatment	Self-administered study questionnaires, medical record, follicular fluid (FF) samples, Society for Assisted Reproductive Technology (SART) guidelines	Reproductive-age women without fertility problems	Association between PFAA in follicular fluid (FF) and the quality of embryo in women undergoing IVF	Increased levels of PFAA in FF are associated with poorer embryo quality during IVF	Exposure to PFAA is associated with adverse outcomes of IVF	5 months follow-up, does not include seminal PFAA concentrations from the male partner, limited generalizability due to IVF patients
Nobles et al. [16]	2023	U.S.A.	Prospective cohort study	1228 women trying to conceive	Blood and urine samples	Reproductive-age women who were not trying to get pregnant	Association of the reproductive effects of phthalates and the ability of women to get pregnant and maintain the pregnancy and also biomarkers of hormone disruption, inflammation, and oxidative stress	Increased levels are associated with decreased fertility, inflammation, oxidative stress and hormonal changes (↓Ε2, ↑LH, ↑FSH at ovulation)	Increased levels are associated with decreased fertility, inflammation, oxidative stress and hormonal changes (↓Ε2, ↑LH, ↑FSH at ovulation)	Up to six menstrual cycles and throughout pregnancy if they became pregnant follow-up, risk of bias related to misclassification of exposure, limited generalizability
Zhang et al. [17]	2021	U.S.A.	Prospective cohort study	386 women in IVF treatment	General and lifestyle questionnaires, anthropometric measurements, urine and blood sample	Reproductive-age women without fertility problems	Association of prenatal urinary phenol concentrations and the risk of preterm birth	High 1st trimester BPS levels and high prenatal BPA levels were associated with increased risk of preterm birth, high 1st trimester benzophenone-3 levels and high 1st and 3rd trimester triclosan levels were inversely associated with risk of preterm birth	Concentrations of BPA in late pregnancy and parabens in early pregnancy are associated with preterm birth	Potential bias due to lack of trimester data, Potential misclassification of chemical exposure due to short half-life, Limited generalizability of findings due to specific sample
Abdo et al. [18]	2023	Jordan	Case–control study	325 women (213 cases with fertility problems)	Interviews, patient medical files, urine samples	Reproductive-age women without fertility problems (95 controls and 16 others)	Levels of phthalates (DEHP) and their metabolites (MEHP: MEHHP, MEOHP) and their role in fertility outcomes	The levels of MEOHP and total DEHP were higher in cases than controls	Positive association of DEHP with sterility/ infertility	Higher number of cases than controls, lack of adjustment for urinary dilution, rapid metabolism and excretion of phthalate compounds
Pan et al. [19]	2019	China	Case–control study	374 women (157 with primary ovarian insufficiency)	Questionnaires, interviews, blood samples	217 healthy women of reproductive age (controls)	Levels of selected persistent organic pollutants (POPs) in the blood and their association with the risk of primary ovarian insufficiency (POI)	Levels of p,p’-DDT were 2.5 to 3 times higher in cases than in controls, sum of p,p′-DDT, its metabolites and PCB were higher in cases than in controls	Increased levels of PCB and p,p′-DDT were associated with high risk of POI	Not reflecting all POP exposure, moderate sample size, risk of bias (for control group)
Pollack et al. [20]	2018	U.S.A.	Cross-sectional study	143 healthy women (509 urine samples)	Questionnaires, anthropometric measurements, urine samples		Association of bisphenol A (BPA), chlorophenols, benzophenones, parabens, and their metabolites with reproductive hormones	Parabens, their metabolites, and BPA were associated with increased estradiol, all factors were associated with increased progesterone, and phenol factor was associated with decreased FSH and LH	Phenols and parabens may influence ovarian hormone levels	Follow-up for up to two menstrual cycles of study, risk of bias related to measurement error, some limitations of PCA method
Gao et al. [21]	2024	China	Cross-sectional study	155 men in IVF therapy	Study of Exposure and Reproductive Health, questionnaires, urine and semen samples	Reproductive-age men without fertility problems	Relationship between EDCs with high levels and semen quality parameters (concentration and motility)	EDCs act negatively on semen quality (motility) and to a minor percentage on sperm quantity (concentration)	EDCs are associated with lower quality of semen parameters and with infertility	Cross-sectional study design, small sample size, potential presence of additional unmeasured confounding factors, examined only 5 main sperm parameters
Rahimi et al. [22]	2020	Iran	Comparative study	645 women (308 greenhouse workers)	Research-made questionnaire, clinical assessment (BMI, BP, PR, RR), blood samples	337 women (housewives)	The effects of working in greenhouse (exposure to pesticides) on reproductive health	Greenhouse workers: high rate of spontaneous abortion, infertility, low birth weight, and preterm birth	Exposure to pesticides can affect fertility and birth outcomes	Lack of evaluation of different types of pesticides used in greenhouse, cross-sectional research method that does not reflect the causal relationship, inability to quantify the amount of pesticides used and to assess the use of protective equipment
Li et al. [23]	2024	China	Prospective cohort study	188 women undergoing assisted reproduction treatment (ART)	Study of Exposure and Reproductive Health (SEARCH), questionnaires, digital health records, and follicular fluid samples	Reproductive-age women without fertility problems	Levels of EDCs in follicular fluid (FF) and their role in oocyte growth and maturation and early reproductive outcomes	EDCs are associated with all four adverse early reproductive outcomes (high quality embryos, mature oocytes, normal fertilized oocytes, and retrieved oocytes)	EDCs can negatively affect reproductive health	Limited generalizability, does not include male factor, and small sample
Palak et al. [24]	2021	Poland	Case–control study	116 men (66 cases with abnormal sperm parameters)	Semen samples, standardized ejaculate examination based on the WHO criteria	50 men with normal sperm (controls)	Association of bisphenol A (BPA) with steroid hormone levels and its role in sperm quality parameters	BPA is related to adverse levels of seminal plasma, total sperm concentration, and normal sperm morphology	BPA negatively affects the male reproductive functions, spermatogenesis, and fertility	Small sample size (may reduce statistical power of the study), limited generalizability
Zhang et al. [10]	2024	China	Prospective case–control study	152 female volunteers (78 cases with decreased ovarian reserve (DOR))	Questionnaires, medical records, biological samples	74 women with non-decreasing ovarian reserve- non-DOR	Relationship between bisphenol S (BPS) and female DOR	Serum BPS levels are related to adverse levels of AMH and E2 and positively related to FSH levels; serum BPA levels are higher in the DOR group than the non-DOR group	BPS and BPA are significant factors for reduced ovarian function and infertility	Small number of cases (limiting conclusions regarding the relationship between BPS exposure and ovarian function), absence of long-term follow-up data of the volunteers (restricts the associations between BPS exposure and reproductive outcomes), lack of information about the crosstalk mechanism of AMH, FSH, LH, and E2 caused by BPS exposure

AMH: Anti-Müllerian Hormone; ART: Assisted Reproductive Technology; BMI: Body Mass Index; BP: Blood Pressure; BPA: Bisphenol A; BPAF: Bisphenol AF; BPP: Bisphenol P; BPS: Bisphenol S; BPZ: Bisphenol Z; DOR: Decreased Ovarian Reserve; DEHP: Di(2-ethylhexyl) Phthalate; E2: Estradiol; EDCs: Endocrine-Disrupting Chemicals; FF: Follicular Fluid; FSH: Follicle-Stimulating Hormone; IVF: In Vitro Fertilization; LH: Luteinizing Hormone; MEHP: Mono(2-ethylhexyl) Phthalate; MEHHP: Mono(2-ethyl-5-hydroxyhexyl) Phthalate; MEOHP: Mono(2-ethyl-5-oxohexyl) Phthalate; PCA: Principal Component Analysis; PCBs: Polychlorinated Biphenyls; PCOS: Polycystic Ovary Syndrome; PFAS: Per- and Polyfluoroalkyl Substances; PFAA: Perfluoroalkyl Acids; POI: Primary Ovarian Insufficiency; POPs: Persistent Organic Pollutants; PR: Pulse Rate; RR: Respiratory Rate; SA: Semen Analysis; SART: Society for Assisted Reproductive Technology; SEARCH: Study of Exposure and Reproductive Health; SHARE: Systematic Harmonization and Research Environment (contextual cohort from Utah); TCC: Triclocarban; TCS: Triclosan; UPDB: Utah Population Database; WHO: World Health Organization.

## Data Availability

All data supporting the findings of this review are derived from sources cited within the article.

## Supplementary Materials

The following supporting information can be downloaded at https://www.mdpi.com/article/10.3390/life15070993/s1, Supplementary Table S1.

## Abbreviations

The following abbreviations are used in this manuscript:

Abbreviation	Definition
AMH	Anti-Müllerian Hormone
ART	Assisted Reproductive Technology
BPA	Bisphenol A
BPAF	Bisphenol AF
BPP	Bisphenol P
BPS	Bisphenol S
BPZ	Bisphenol Z
BMI	Body Mass Index
BP	Blood Pressure
DDT	Dichlorodiphenyltrichloroethane
DEHP	Di(2-ethylhexyl) Phthalate
DOR	Decreased Ovarian Reserve
E2	Estradiol
EDCs	Endocrine-Disrupting Chemicals
ERRγ	Estrogen-Related Receptor Gamma
ERα	Estrogen Receptor Alpha
ERβ	Estrogen Receptor Beta
FF	Follicular Fluid
FSH	Follicle-Stimulating Hormone
GPER	G Protein-Coupled Estrogen Receptor
IVF	In Vitro Fertilization
LH	Luteinizing Hormone
MEHP	Mono(2-ethylhexyl) Phthalate
MEHHP	Mono(2-ethyl-5-hydroxyhexyl) Phthalate
MEOHP	Mono(2-ethyl-5-oxohexyl) Phthalate
PCA	Principal Component Analysis
PCBs	Polychlorinated Biphenyls
PCOS	Polycystic Ovary Syndrome
PFAA	Perfluoroalkyl Acids
PFAS	Per- and Polyfluoroalkyl Substances
PFCs	Perfluorinated Compounds
POI	Primary Ovarian Insufficiency
POPs	Persistent Organic Pollutants
PR	Pulse Rate
PROSPERO	International Prospective Register of Systematic Reviews
RR	Respiratory Rate
SA	Semen Analysis
SART	Society for Assisted Reproductive Technology
SEARCH	Study of Exposure and Reproductive Health
SHARE	Systematic Harmonization and Research Environment
TCC	Triclocarban
TCS	Triclosan
UPDB	Utah Population Database
WHO	World Health Organization
