# Supplementary material for: Associations Between Endocrine-Disrupting Chemical Exposure and Fertility Outcomes: A Decade of Human Epidemiological Evidence
Source: Life (Basel). 2025 Jun 21;15(7):993. doi: 10.3390/life15070993 (PMC12299029; doi:10.3390/life15070993)
Supplement: Supplementary file 1 [file life-15-00993-s001.zip › life-3692064-supplementary.pdf]

Supplementary Table 1. Caldwell framework

Authors	Year	Country	Study Design	Sample size	Measurement methods	Data Analysis	Limitations
Ramsay et al. [13]	2023	U.S.A.	Retrospective cohort study	21.563 men dealing with fertility problems (23.922 samples)	SHARE cohort (all semen analysis results 1996-2017 in Utah), Utah Population Database (UPDB)	4th and 5th edition of the WHO manual for examination and processing of human semen, Risk-Screening Environmental Indicators Geographic Microdata (RSEI-GM)-to compare chemical levels and trends around reporting facilities and afterwards the exposure to airborne EDCs from industrial sources, multivariable regression models with robust standard errors (association of EDCs and semen parameters)	Limited generalizability due to the sample of infertile men, No information about the occupation, No adjustment for any comorbid medical conditions, Lack of diversity
Zhan et al. [9]	2023	China	Retrospective Case-Control Study (multi-center, hospital-based)	733 women (321 women with PCOS-412 women with male infertility)	Questionnaires, blood, urine, semen samples	Spearman Correlation (evaluation of bivariate correlations between bisphenol analogs), Spearman Correlation and Generalized Linear Regression (evaluate the association between bisphenol analogs and hormonal parameters), multiple logistic regression (examine the association between each bisphenol compound and the odds of PCOS), restricted cubic spline (RCS) model (identify the potential nonlinear relationship between urinary bisphenol compounds and the odds of PCOS), quantile-based g computation (QGC) (for the overall association of bisphenol analogs), sensitivity analyses (for the consistency of the results)	Exposure to BPA was measured after the diagnosis of PCOS, Bisphenol analogs have rapid elimination from the body and short half-lives, No information on dietary habits (healthy diet can reduce the odds of PCOS)
Wei et al. [14]	2024	China	Case-Control Study	302 women (181 cases with fertility problems, 121 controls)	Blood samples, clinical diagnosis	Binary Logistic Regression (association between serum levels of EDCs and the risk of infertility), Random	Lack of rigorous inclusion criteria, single sample measurement, Need of animal

						Forest Model (analysis of the relative importance of EDCs), Out-of-bag (OOB) error, accuracy, and AUC (to assess the stability and performance of the random forest models), Odds ratios (ORs) and their corresponding 95% confidence intervals (CI) (risk factors for infertility)	experiments to reinforce the findings
Zeng et al. [15]	2023	China	Prospective Cohort Study	729 women in IVF therapy	Self-administered study questionnaires, medical record, follicular fluid (FF) samples, Society for Assisted Reproductive Technology (SART) guidelines	Ultra-performance liquid chromatography coupled with triple quadrupole tandem mass spectrometry with electrospray ionization in a negative mode (analysis of 32 PFAA's concentrations in FF), Log-binominal regression models (estimate associations between the presence of ≥1 high-quality embryo and the concentrations of PFAA in FF), CAUSALMED procedure in SAS (to investigate oocyte maturity and explain the association between PFAA exposure and embryo quality), Bayesian kernel machine regression (to estimate the potential effect of a PFAA mixture on embryo quality), Restricted cubic splines (RCS) (for linearity of associations), Multivariable linear regression models (estimate the association between PFAA levels in FF and high-quality embryo rate), RCS model(to calculate how the different levels of PFAA in FF can affect the high-quality embryo rate)	Not include seminal PFAA concentrations from the male partner, Limited generalizability due to IVF patients
Nobles et al. [16]	2023	U.S.A.	Prospective Study	1.228 women undergoing IVF	Blood and urine samples	Enzymatic deconjugation followed by solid-phase extraction and liquid chromatography with tandem mass	Risk of bias related to misclassification of exposure,

						spectrometry (to measure phthalates), Spearman correlation coefficients (association between phthalate metabolites), Poisson regression (for risk of pregnancy loss), generalized linear models (influence of phthalate metabolites on the menstrual cycle), Benjamini–Hochberg procedure (to correct the false discovery rate)	Limited generalizability
Zhang et al. [17]	2021	U.S.A.	Prospective Cohort Study	386 women in IVF treatment	General and lifestyle questionnaires, anthropometric measurements, urine and blood samples	Spearman correlation coefficients (assess the correlations between measures of the same phenol biomarker concentrations throughout trimesters), intraclass correlation coefficients (assess the temporal variability of the prenatal phenol concentrations), directed acyclic graph (selection of covariates)	Potential bias due to lack of trimester data, potential misclassification of chemical exposure due to short half-life, Limited generalizability of findings due to specific sample
Abdo et al. [18]	2023	Jordan	Case-Control Study	325 women (213 cases with fertility problems, 95 controls and 16 others)	Interviews, patient medical files, urine samples	Parametric T-test and Non-parametric Wilcoxon test (for differences in the means and medians of cases and controls for each metabolite), Chi square tests (for differences in sociodemographic characteristics between cases and controls, Pearson and Spearman correlation coefficients matrixes (for assess the relationships between metabolite concentrations, Multivariate logistic regression (for candidate covariates)	Higher number of cases than controls, Lack of adjustment for urinary dilution, Rapid metabolism and excretion of phthalate compounds
Pan et al. [19]	2019	China	Case-Control Study	374 women (157 cases 157 with primary ovarian insufficiency	Questionnaires, interviews, blood sample	Mann-Whitney U test (to compare POPs levels between cases and controls), Binary logistic regression models (for dose-response relationship between	Not reflect all POPs exposure, Moderate sample size, Risk of bias (for control group)

				and 217 controls)		POPs and POI), Multiple linear regression model (to analyze the correlations of PCA predictor variables with hormones in both groups)	
Pollack et al. [20]	2018	U.S.A.	Cross-Sectional Study	143 women (509 urine samples)	Questionnaires, anthropometric measurements, urine samples	Liquid chromatography coupled with API2000 electrospray triple-quadrupole mass spectrometry (sample analysis and quantification of phenols and parabens), solid-phase competitive chemiluminescent enzymatic immunoassay (to measure estradiol, progesterone, LH, FSH) and single chemical linear mixed models (to determine their associations with BPA, chlorophenols, benzophenones and parabens), principal component analysis (PCA) (for association between multiple chemical exposures and hormones)	Risk of bias related to measurement error, Some limitations of PCA method
Gao et al. [21]	2024	China	Cross-Sectional Study	155 men in IVF therapy	'Study of Exposure and Reproductive Health'', questionnaires, urine and semen samples	Automated semen analysis machine coupled with analytical software (to measure semen parameters), WHO standards (to assess semen quality), liquid chromatography with triple quadrupole mass spectrometry (to detect EDCs), QA/QC procedures (for the consistency and reliability of the measurements), Chi-square test and Kruskal-Wallis test (for differences in demographic characteristics of participants), Spearman rank correlation (for intercorrelation between EDCs), multivariable linear and logistic	Cross-sectional study design, Small sample size, Potential presence of additional unmeasured confounding factors, Examine only 5 main sperm parameters

						regressions (to assess the concentration of EDCs and semen quality parameters), Bayesian Kernel Machine Regression (BKMR) (for the relationship between exposure and outcome), quantile-G-computation (QGC) analysis (to estimate the joint distribution)	
Rahimi et al. [22]	2020	Iran	Comparative Cross-Sectional Study	645 women (308 greenhouse workers)	Research-made questionnaire, clinical assessment (BMI, BP, PR, RR), blood samples	Kumologov-Smirnov test (to check data for normality), non-parametric tests (for further analysis), Mann-Whitney test (for assessment variables), Chi-square test and Fisher's exact test (to compare the differences in the demographic data between the groups), gender-matched interviewers (to reduce the information bias)	Lack of evaluation of different types of pesticides used in greenhouse, Cross-sectional research method that does not reflect the casual relationship, Inability to quantify the amount of pesticides used and to assess the use of protective equipment
Li et al. [23]	2024	China	Prospective Cohort Study	188 women undergoing assisted reproductio n treatment (ART)	Study of Exposure and Reproductive Health (SEARCH), questionnaires, digital health records, follicular fluid samples	Spearman correlation analysis (for correlations between follicular fluid chemicals), Poisson models (for relationships between level of individual contaminants and outcomes), Benjamini-Hochberg false discovery rate (FDR) (to adjust the results of multiple hypothesis testing), Bayesian Kernel Machine Regression (BKMR) (to investigate the effects of the EDCs mixture in follicular fluid on the outcomes)	Limited generalizability, Not include male factor, Small sample size
Palak et al. [24]	2021	Poland	Case-Control Study	116 men (66 cases with abnormal sperm parameters and 50 controls with normal	Semen samples, standardized ejaculate examination based on the WHO criteria	Computer-aided semen analysis in accordance with WHO guidelines (2010), Diff-Quik staining set (to evaluate sperm morphology), Spearman's correlation coefficient (for associations between the	Small sample size (may reduce statistical power of the study), Limited generalizability

				sperm parameters)		studied parameters), Kruskal-Wallis test with Dunn's post hoc test (due to non-normal distribution of data), GraphPad Prism 9 (for statistical analyses)	
Zhang et al. [10]	2024	China	Prospective Case-Control Study	152 female volunteers (78 cases with decrease ovarian reserve (DOR) and 74 controls with non-decreased ovarian function (non-DOR))	Questionnaires, medical records, biological samples	Enzyme-linked immunosorbent assay (ELISA) (to quantify BPS serum levels of volunteers), Linear regression models and logistic regression models (for association between BPS levels and DOR indicators), Restricted cubic spline (RCS) models (to explore potential non-linear associations), Spearman rank correlation (for correlation analysis), Kolmogorov-Smirnov test (for the state of distribution of the data)	Small number of cases (limiting conclusions regarding the relationship between BPS exposure and the ovarian function), Absence of long-term follow-up data of the volunteers (restricts the associations between BPS exposure and reproductive outcomes), Lack of information about the crosstalk mechanism of AMH, FSH, LH and E2 caused by BPS exposure

AMH: Anti-Müllerian Hormone, API: Atmospheric Pressure Ionization, ART: Assisted Reproductive Technology, AUC: Area Under the Curve, BKMR: Bayesian Kernel Machine Regression, BMI: Body Mass Index, BP: Blood Pressure, BPS: Bisphenol S, CI: Confidence Interval, DOR: Decreased Ovarian Reserve, EDCs: Endocrine-Disrupting Chemicals, ELISA: Enzyme-Linked Immunosorbent Assay, E2: Estradiol, FDR: False Discovery Rate, FF: Follicular Fluid, FSH: Follicle-Stimulating Hormone, GM: Geographic Microdata, IVF: In Vitro Fertilization, LH: Luteinizing Hormone, OR: Odds Ratio, OOB: Out-of-Bag, PCA: Principal Component Analysis, PCOS: Polycystic Ovary Syndrome, POI: Primary Ovarian Insufficiency, POP: Persistent Organic Pollutant, PR: Pulse Rate, QGC: Quantile-Based g Computation, QA/QC: Quality Assurance / Quality Control, RCS: Restricted Cubic Spline, RR: Respiratory Rate, RSEI-GM: Risk-Screening Environmental Indicators – Geographic Microdata, SART: Society for Assisted Reproductive Technology, SEARCH: Study of Exposure and Reproductive Health, SHARE: Subfertility Health and Assisted Reproduction and the Environment, UPDB: Utah Population Database, WHO: World Health Organization
